# The Synthetic Tie2 Agonist Peptide Vasculotide Protects Renal Vascular Barrier Function In Experimental Acute Kidney Injury

**DOI:** 10.1038/srep22111

**Published:** 2016-02-25

**Authors:** Eva Rübig, Jörg Stypmann, Paul Van Slyke, Daniel J Dumont, Tilmann Spieker, Konrad Buscher, Stefan Reuter, Tobias Goerge, Hermann Pavenstädt, Philipp Kümpers

**Affiliations:** 1 Department of Medicine D, Division of General Internal Medicine, Nephrology, and Rheumatology, University Hospital Münster, Albert-Schweitzer-Strasse 33, 48149 Münster, Germany; 2 Department of Cardiology, University Hospital Münster, Albert-Schweitzer-Strasse 33, 48149 Münster, Germany; 3 Vasomune Therapeutics, 661 University Avenue Suite 465, Toronto, Ontario, Canada; 4 Molecular and Cellular Biology Research, Sunnybrook Research Institute, and Department of Medical Biophysics, University of Toronto, Ontario, Canada; 5 Institute of Pathology at St Franziskus Hospital Münster, Hohenzollernring 64, 48145 Münster, Germany; 6 Department of Dermatology, University Hospital Münster, Von-Esmarch-Straße 58, 48149 Münster, Germany

## Abstract

Microvascular barrier dysfunction plays a major role in the pathophysiology of acute kidney injury (AKI). Angiopoietin-1, the natural agonist ligand for the endothelial-specific Tie2 receptor, is a non-redundant endothelial survival and vascular stabilization factor. Here we evaluate the efficacy of a polyethylene glycol-clustered Tie2 agonist peptide, vasculotide (VT), to protect against endothelial-cell activation with subsequent microvascular dysfunction in a murine model of ischemic AKI. Renal ischemia reperfusion injury (IRI) was induced by clamping of the renal arteries for 35 minutes. Mice were treated with VT or PEGylated cysteine before IRI. Sham-operated animals served as time-matched controls. Treatment with VT significantly reduced transcapillary albumin flux and renal tissue edema after IRI. The protective effects of VT were associated with activation of Tie2 and stabilization of its downstream effector, VE-cadherin in renal vasculature. VT abolished the decline in renal tissue blood flow, attenuated the increase of serum creatinine and blood urea nitrogen after IRI, improved recovery of renal function and markedly reduced mortality compared to PEG [HR 0.14 (95% CI 0.05–0.78) *P* < 0.05]. VT is inexpensive to produce, chemically stable and unrelated to any Tie2 ligands. Thus, VT may represent a novel therapy to prevent AKI in patients.

Renal ischemia, due to hypotension, hypoperfusion and/or systemic inflammation, is the major cause of acute kidney injury (AKI), a relatively common clinical syndrome that is associated with high morbidity and mortality in critically ill patients^1^,^2^. While tubular epithelial cell injury has long been of central importance in explaining the pathophysiology of AKI, this paradigm has recently evolved to include the vascular endothelium, an organ that regulates blood flow and modulates coagulation, inflammation and vascular permeability. Independent of the causative factors, AKI has deleterious effects on the renal vascular endothelium resulting in microvascular dysfunction leading to increased microvascular permeability, vascular congestion and renal edema^3^,^4^,^5^. Together with other endothelium-dependent events such as vasoconstriction, activated coagulation and enhanced adherence of inflammatory cells to the endothelium, renal edema formation impairs microvascular blood flow which eventually culminates in an intrinsic renal compartment syndrome^6^,^7^.

Recently, the endothelial Angiopoietin-Tie2 ligand-receptor system has been recognized as a non-redundant signalling pathway that controls vascular inflammation and permeability, key features of early AKI. The vascular-limited receptor tyrosine kinases Tie2 and its agonist ligand Angiopoietin-1 (Angpt1) were discovered in the mid-1990 s by Sato^8^ and Davis^9^. While early studies in Angpt1−/− and Tie2 −/− knockout mice, which die in utero owing to severe vascular defects, revealed the importance of operational Angpt1-Tie2 signalling for developmental angiogenesis^8^,^9^,^10^, Angpt1 was subsequently identified as a transdominant anti-permeability factor that protects the vasculature of adult mice from plasma leakage induced by vascular endothelial growth factor (VEGF) and other inflammatory stimuli^11^,^12^. In contrast, release of Angiopoietin-2 (Angpt2) from endothelial Weibel-Pallade bodies disrupts the constitutive Angpt1-Tie2 signaling by preventing Angpt1 from binding to the receptor^13^,^14^ thereby promoting inflammation and permeability^15^,^16^,^17^.

Recent studies in animals and humans have demonstrated that renal ischemia induces a transient imbalance between renal Angpt1 und -2 expression in favour of Angpt2^18^,^19^. During reperfusion renal vascular endothelium acutely releases pre-stored Angpt2^19^. Given the absence of redundant systems to bypass the function of Tie2, it was speculated that exogenous repletion of Angpt1 rescues Tie2 signalling and thus effectively abolishes microvascular leakage in ischemia-reperfusion injury (IRI). Indeed, overexpression of an engineered Angpt1 variant in mice attenuates renal IRI^20^ and pretreatment of donor hearts with this Angpt1 variant^21^ or with an inhibitor of Angpt2^22^ improves IRI after heart transplantation in rats. Thus, pharmaceutical agents that activate the Tie2 receptor pathway leading to vascular stabilization and protection against microvascular dysfunction in AKI would be highly desirable. In this study we tested the effects of the novel Tie2-agonist tetrameric peptide, Vasculotide (VT)^23^,^24^ in a murine model of renal IRI.

## Results

### Vasculotide activates Tie2 signalling and protects vascular barrier in ischemic kidneys

**Figure 1:**
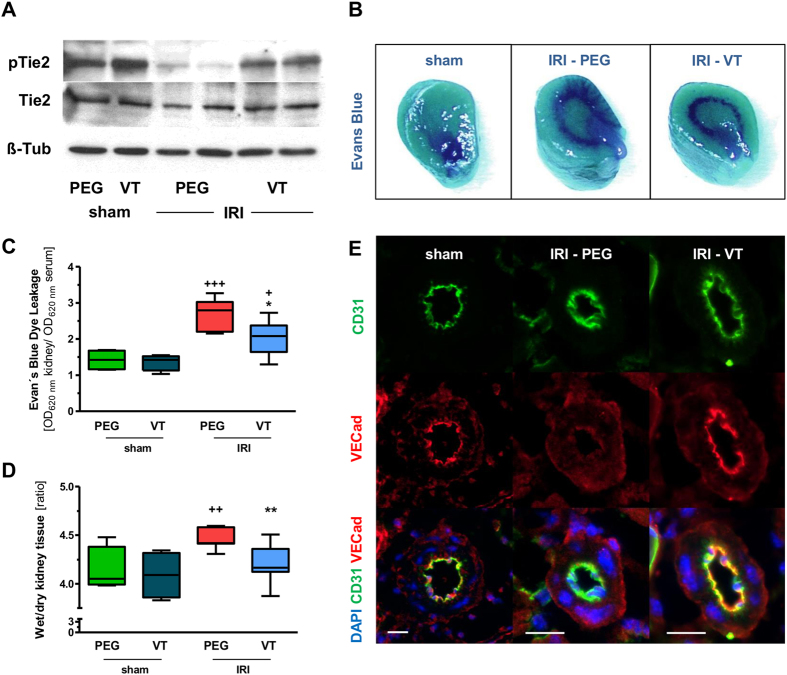
Effects of Vasculotide on Tie2 signaling, capillary leakage and endothelial integrity. Mice (n = 8 per group) were pre-treated with 200 ng of Vasculotide (VT) or pegylated cystein (PEG) i.p. at −16 h, −1 h prior to renal ischemia reperfusion injury (IRI) or sham surgery. **(A)** Immunoblots for phosphoTie-2Y992 (pTie2) and total Tie2 (Tie2) in kidney homogenates obtained 24 h after renal IRI. VT: Vasculotide; PEG: PEG-Cys (control); ß-Tub: ß-Tubulin. **(B)** Macroscopic aspect of Evans blue dye in kidneys, **(C)** Evans blue dye content and **(D)** Wet-to-dry-ratio in kidney homogenates 24 h after IRI or sham operation. OD: optical density. **(E)** Immunofluorescence for VE-Cadherin and the endothelial marker CD31 at 24 h after IRI or sham operation. VECad: VE-Cadherin. Data are means ± SEM. *P < 0.05; **P < 0.01 vs. IRI + PEG-Cys. ^+^*P* < 0.05; ^*++*^*P* < 0.01; ^+++^*P* < 0.001 vs. sham.

Figure 1 shows the VT (Supplementary Fig. S1) induced changes of Tie2 expression and phosphorylation in kidney homogenates 24 h after IRI. Mice received 200 ng VT (or molar equivalent of PEGylated cysteine (hereafter PEG)) intraperitoneally (i.p.) at −16 h and at −1 h prior to IRI or sham surgery, respectively. VT pretreatment almost completely prevented Tie2 de-phosphorylation (Fig. 1A). To resolve the impact of the changes in Tie2 activation we next surveyed vascular leakage in the kidney of these mice. The anti-leakage effect of VT on the kidney after IRI was already macroscopically visible (Fig. 1B). IRI caused a significant increase in transcapillary albumin flux (*P* < 0.001) which was substantially ameliorated by VT (*P* < 0.05) (Fig. 1C). VT treatment completely prevented IRI-induced edema formation in the kidneys (*P* < 0.01) (Fig. 1D). We further evaluated the renal expression of VE-cadherin, a component of endothelial cell-to-cell adherens junctions, whose homotypic interaction between adjacent endothelial cells is essential for the maintenance of vascular barrier function and integrity. VE-cadherin staining in renal endothelium (confirmed by CD31 costaining) was markedly attenuated by IRI and restored to normal by VT pretreatment (Fig. 1E).

### Vasculotide protects renal microcirculation but not tubular damage

**Figure 2:**
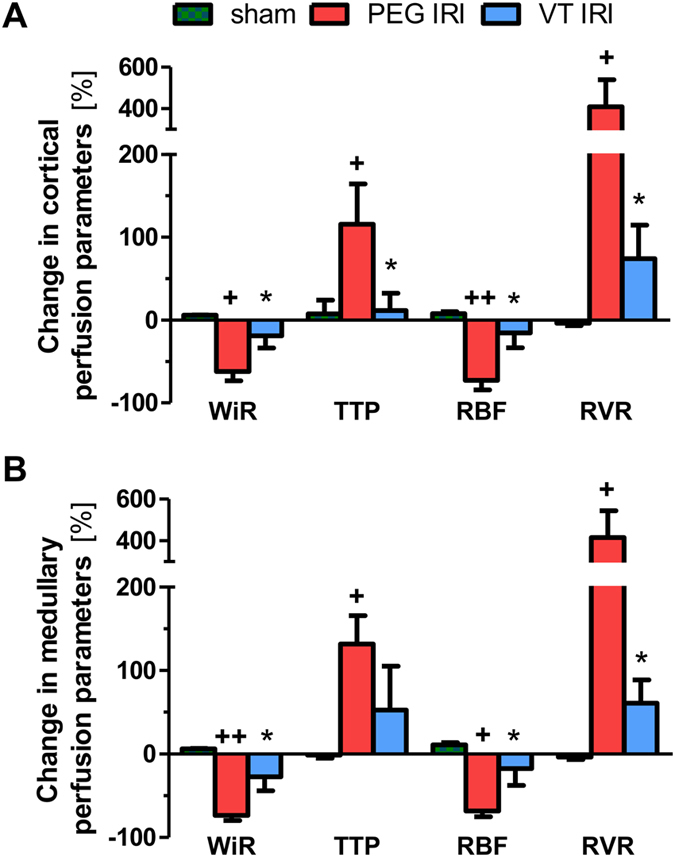
Vasculotide improves renal perfusion. Mice were pre-treated with 200 ng of VT or PEG i.p. at −16 h and −1 h prior to renal IRI (n = 7 per group) or sham surgery (n = 4 per group). Change of cortical **(A**) and medullary **(B)** perfusion parameters (contrast enhanced ultrasound) 1 h after renal IRI. Every mouse was examined immediately before and 1 h after IRI or sham operation. Renal vascular resistance 1 h after IRI was calculated as follows: RVR = MAP/RBF. VT: Vasculotide; PEG: PEG-Cys (control); WiR: wash in rate; TTP: time to peak; RBF: renal blood flow; RVR: renal vascular resistance. Data are expressed as means ± SEM. *P < *0.05* vs. IRI + PEG-Cys. ^*+*^*P* < 0.05; ^*++*^*P* < 0.01 vs. Sham.

Having observed considerably less leakage and edema formation in ischemic kidneys from VT-treated animals (Fig. 1) we next used contrast-enhanced ultrasound (CEUS) to visualize and quantify VT´s effect on renal perfusion. CEUS uses contrast agents, which are composed of microbubbles of an injectable gas in a supporting shell (phospholipids or proteins)^25^,^26^. Their size (1 to 6 μm), similar to red blood cells, allows microbubbles to cross the capillary bed of the pulmonary circulation and to flow up to capillary level of the kidney, enabling assessment of its microcirculation. CEUS-derived parameters and methodology are explained in Table 1 and Supplementary Fig. S2, respectively. Serial CEUS was performed immediately before and 1 h after renal IRI or sham surgery. While changes were virtually absent in sham controls, IRI altered perfusion parameters (see Table 1 for details) in a consistent and robust fashion. As shown in Fig. 2A,B, we found a decrease in relative blood flow measures (wash in rate: WiR, relative blood flow: rBF) paralleled by longer time-to-peak (TTP) and an increase in relative renal vascular resistance (rRvR) in both cortex and medulla (all *P* < 0.05 vs. Sham). VT pretreatment almost completely prevented these changes in cortical and medullary perfusion parameters (all *P* < 0.05 vs. PEG/IRI and *P* = not significant vs. Sham) (Fig. 2A,B). Time-matched recordings of blood pressure and heart rate showed constant values across all groups and time-points (Table 2). These data suggest that VT-mediated prevention of IRI-induced deterioration of intra-renal microcirculation occurs independently of changes in systemic hemodynamics.

**Figure 3:**
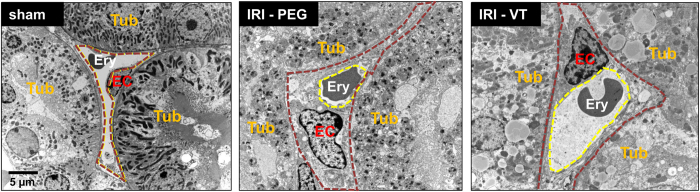
Ultrastructure of peritubular capillaries. Representative transmission-electron microscopy images of peritubular capillaries. Tub = tubular epithelial cell; EC = nucleus of endothelial cell; Ery = erythrocyte; the brown dotted lines indicate the anatomical dimension, the yellow dotted lines the effective luminal dimension of the capillaries. VT: Vasculotide; PEG: PEG-Cys (control); IRI: ischemia reperfusion injury.

Finally, transmission electron microscopy images of peritubular capillaries showed vacuolar degeneration of the endothelial cells as well as thickening and multilayer basement membrane formation as a result of renal IRI. Pretreatment with VT attenuated these alterations and sustained endothelial integrity (Fig. 3).

**Figure 4:**
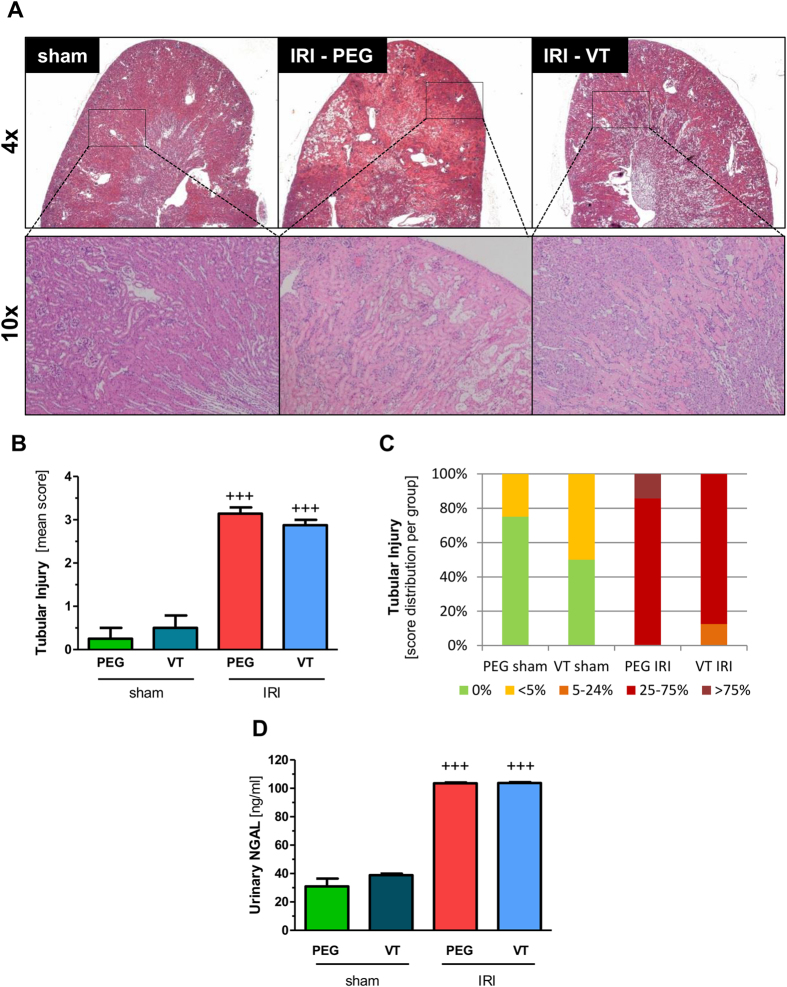
Vasculotide does not prevent acute tubular injury. Mice (n = 8 per group) were pre-treated with 200 ng of VT or PEG i.p. at −16 h, −1 h prior to renal IRI or sham surgery. **(A)** Representative H&E stained kidney sections, **(B**) mean tubular injury score and **(C)** distribution of tubular injury severity (PEG-IRI vs VT-IRI: Chi-square test P = 0.3) between the groups 24 h after IRI or sham surgery. **(D)** Levels of urinary NGAL 24 h after renal IRI. Data are means ± SEM. ^+++^*P* < 0.001 vs. sham.

We next asked whether protection of the renal vasculature by VT had an effect on tubular damage. Illustrative hematoxylin-eosin images are shown in Fig. 4A. As expected, ischemic kidneys showed moderate-to-severe tubular injury, characterized by epithelial necrosis and tubular dilatation in the cortex or the outer medulla. VT, however, did not substantially improve mean tubular injury score or damage pattern (Chi-square test P = 0.3), respectively (Fig. 4B,C). To further confirm these findings we quantified urinary neutrophil gelatinase associated lipocalin (NGAL), a novel marker of tubular damage that is proportional to the severity of AKI^27^,^28^. Consistent with the histologic data, NGAL levels were not different between the PEG and the VT pretreated IRI groups (Fig. 4D).

### Vasculotide improves kidney function and reduces mortality after renal IRI

**Figure 5:**
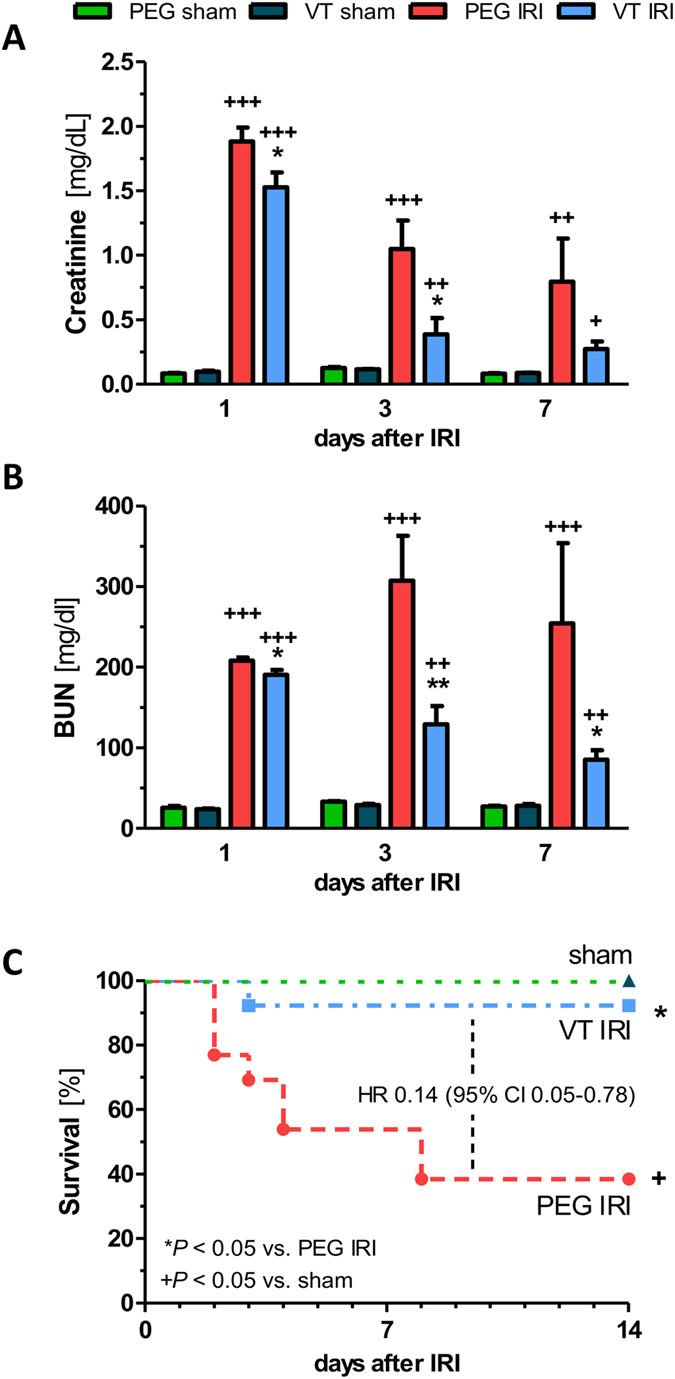
Vasculotide improves kidney function and reduces mortality after renal ischemia reperfusion injury. Serum levels of **(A)** creatinine and **(B)** blood urea nitrogen (BUN) at day 1, 3 and 7 after renal IRI (n = 8 per group) or sham surgery (n = 4 per group). **(C)** survival after pretreatment with 200 ng VT or PEG −16 h and −1 h before renal IRI as well as +24 h after surgery (n = 13 per group) or sham operation (n = 4). Data are expressed as means ± SEM. *P < 0.05; **P < 0.01 vs. IRI + PEG-Cys. ^+^*P* < 0.05; ^*++*^*P* < 0.01; ^+++^*P* < 0.001 vs. sham. HR: Hazard Ratio, CI: Confidence Interval.

Bilateral IRI resulted in a significant increase in serum creatinine and blood urea nitrogen (BUN) levels in PEG-treated controls. Treatment with VT was sufficient to attenuate ischemic AKI, as evidenced by significantly lower serum creatinine and BUN levels in VT-treated mice compared to controls (*P* < 0.05), especially at later time points (day 3 and day 7 after IRI) (Fig. 5A,B and Supplementary Fig. S3).

Lastly, we performed survival experiments, the benchmark of any successful treatment regimen. Mice received an additional dose of 200 ng VT (or molar equivalent PEG) i.p. 24 h post to sham or surgery in addition to standard VT-pretreatment. As a result, only 38% of mice pre-treated with PEG survived, compared to 92% in the VT group, respectively. When compared to the PEG group, pre-treatment with VT was significantly associated with better outcome [Hazard Ratio 0.14 (95% Confidence interval 0.05–0.78) *P* < 0.05] (Fig. 5C).

## Discussion

Although AKI is a frequent complication in the intensive care unit (ICU) that is associated with increased mortality and morbidity, no specific treatments are available^29^. Current understanding of the pathophysiology of AKI emphasizes the complex interplay between inflammation and ischemia-reperfusion leading to microcirculatory dysfunction and localized hypoperfusion and ischemia of the kidney^2^,^4^,^5^. Here we used a reliable model of AKI to study the impact of VT on kidney function and mortality. We show that VT counteracts microvascular barrier dysfunction of the renal microcirculation in renal IRI. VT activated Tie2 and its major downstream pathway VE-cadherin in the kidney, prevented IRI-induced renal vascular leakage and congestion, improved renal function and recovery, and significantly attenuated mortality after renal IRI. These effects were independent of changes in systemic hemodynamics and tubular injury. Together, the present data suggest that Tie2 activation by VT is sufficient to preserve endothelial barrier function and to confer protection against ischemic AKI.

We and others have shown that Tie2 protein abundance, Tie2 activation, and downstream signaling are suppressed in murine lungs and kidneys during endotoxic and hemorrhagic shock^23^,^30^,^31^. Recently David *et al.* demonstrated that VT is able to overcome the decreased Tie2 protein abundance during endotoxemia by increased activation of the remaining Tie2 molecules^32^. Tie2 expression, however, has not been addressed in renal IRI. We found marked loss of phosphorylated Tie2 in kidneys subjected to IRI. As in septic lungs^32^, VT was able to increase Tie2 phosphorylation despite loss of total Tie-2 protein, thereby preserving net flux through this signaling pathway. In a proof-of-principle study, Jung *et al.*^20^ demonstrated that adenoviral overexpression of cartilage oligomeric matrix protein-angiopoietin-1 (COMP-Angpt1), an engineered variant of native Angpt1 with higher activity, attenuates renal IRI in mice. Although the current study basically corroborates their findings^20^, some contrasting results deserve further discussion. First, overexpression of COMP-Angpt1 ameliorated tubular injury, whereas VT seemed to have no significant tubulo-protective effect, as shown by histology and urinary NGAL. Given that tubuli do not express Tie2^33^, the latter finding is not surprising. However, an explanation for this discrepancy could be that COMP-Angpt1 is capable of mediating some protective effects independently of Tie2, possibly through its integrin-binding sequence QHREDGS that VT lacks. Such an integrin-β1 dependent suppression of hypoxia-induced apoptosis by Angpt1 has already been established in cardiomyocytes, which lack Tie2 as well^34^. Another possibility to account for the presumable distinctions between COMP-Angpt1 and VT is that higher severity of AKI (22 vs. 35 min clamp time in the current study) may preclude detection of subtle tubular protection conferred by VT. In line with this notion, Jung *et al.*^20^ detected only small histopathologic distinctions despite rather huge differences in serum creatinine values. In fact we too observed a trend towards less tubular damage in VT-treated mice. According to the steeper decline of creatinine and BUN at day 3 and 7, it is conceivable that this difference might become significant at later time points.

Second, in contrast to the aforementioned study by Jung *et al.* we clamped not only the renal artery, but the whole renal pedicle, which has been shown to reduce renal blood flow even further^35^. They used standard invasive laser-Doppler flowmetry to assess renal blood flow in kidneys after IRI. CEUS yielded results similar to laser-Doppler flowmetry in murine kidneys treated with increasing doses of the vasoconstrictor Endothelin-1, supporting the validity of this non-invasive technique^36^. We performed repeated CEUS measurements in a single mouse (pre- and post-surgery) in exactly the same position of animal, transducer and recording target region. Together these differences resulted in a stronger decline in medullary blood flow (−30 vs. −60%) shortly after reperfusion in our model. We further noted a marked decline in cortical blood flow (−0% vs −50%), owing to the higher severity of our model – which was tailored to result in a mortality of >50% in the control group. It is thus remarkable, that VT completely prevented IRI-induced changes in both, medullary and cortical perfusion parameters. This appeared to be mediated independent of changes in systemic hemodynamics, which argues against improved renal blood flow as mere consequence of better blood pressure. Herrler *et al.* recently uncovered that edema-related pressure elevation in the ischemic kidney can last for several days and accounts for impaired renal perfusion in AKI^6^,^7^. They conclusively demonstrated that surgical pressure relief - via perforation of the renal capsule – prevented any reduction in renal blood flow after renal IRI^7^. We therefore speculate that the improvement in renal perfusion seen with VT treatment is mainly due to reduced capillary leakage and subsequent formation of renal tissue edema after IRI. Mechanistically, Tie2 stimulation counteracts (hyper-)permeability through multi-level effects on intracellular signaling, cytoskeleton and junction-related molecules^37^,^38^,^39^. In accordance with previous *in vitro* and *in vivo* studies we observed stabilization of the particularly important adherens-junction protein VE-cadherin, a well-defined downstream target of Tie2, conferred by VT^15^,^32^.

We also analyzed survival after ischemic AKI. Probability of death from ischemic AKI was 86% lower in the VT-pretreated group [HR 0.14 (95% CI 0.05–0.78)]. Recent evidence suggests, that not only primary ischemic damage to the kidney, but also secondary, so-called remote organ injury, may affect outcome in AKI^40^. Given that Tie2 mRNA and protein are most abundant in the pulmonary vasculature, it is conceivable that the lung is not only uniquely dependent on Tie2 signaling to maintain endothelial integrity, but that it may also constitute a target for deleterious Angpt2 released from the ischemic kidney during reperfusion. At least pulmonary vascular-specific Tie2 gene expression has already been shown to decline in response to renal IRI^41^. It is thus conceivable, that part of the survival benefit in our model depends on a remote-organ “benefit” due to Tie2-stimulation by VT.

Our data suggest that selective protection of microvascular barrier function is sufficient to improve renal recovery and outcome in renal IRI. A Tie2 agonist drug may therefore have great potential as a vascular barrier-protective agent in human AKI. The potential clinical use of recombinant Angpt1, the endogenous ligand for Tie2, is limited by its short protein half-time, an inherent predisposition to protein aggregation, poor solubility of the protein, and the costly production^42^. Due to these limitations the recombinant form of human Angpt1, and actually its improved variant COMP-Angpt1, represent considerable challenges for use in AKI patients. In contrast, VT was invented based on phage display experiments in which over 1 billion unique peptides were screened to identify those capable of binding Tie2 with high affinity but outside of the shared Angpt1/2 binding pocket^24^,^43^. Vasculotide bears no sequence homology to Angpt1, a fact that negates the potential of an individual to develop autoantibodies to endogenous Angpt1 or components of the chimeric protein, COMP-Angpt1. Preliminary work also demonstrates that VT neither activates, nor inhibits cellular receptors other than Tie2 (Supplementary Fig. 4, Supplementary Table 1, 2). In previous work, we have observed that a single dose of VT (200 ng per mouse i.p.) can sustain Tie2 phosphorylation in kidneys for >24 h^23^. Pretreatment with the same dose protected vascular barrier function in mouse models of acute skin radiation damage^44^ and septic multiple organ failure^23^, while a slightly higher dose (500 ng per mouse) was used in models of endotoxemic lung injury^32^ and lethal influenza^45^. Given that loss of Tie2 signalling (via loss of Tie2 and/or binding of released Angpt2) seems to be an early event in AKI, it seems to be exceedingly important that VT is given as soon as possible in order to maximize protective signaling through the remaining pool of Tie2 receptors. In line with this notion, a VT-rescue approach (i.e. VT first administered 2 h after reperfusion) still resulted in improved survival compared to PEG [HR 0.25 (95% CI 0.09 – 0.72) P < 0.01)] but differences between groups were modest (Supplementary Fig. S5). However, because of the immediate and strong decline of renal blood flow (i.e. functional dropout of impaired capillaries) in IRI, any drug delivered through the bloodstream at 2 h after the insult may not have full access to its (pharmacological) target (as with pre-treatment). Therefore, post-treatment testing in this specific severe model of renal IRI represents a formidable treatment challenge. From a clinical perspective, endothelium-targeted (pre-)treatment with VT could represent a feasible approach to preventing AKI in the setting of radio contrast administration, renal transplantation and major surgery in high-risk patients (including coronary artery bypass graft). Further studies are needed to identify optimal dosing regimens, improve understanding of prophylactic vs. therapeutic (i.e. post injury) VT administration and to examine the long term impact of (specifically) improving the vascular component of AKI. As mentioned above, VT did not prevent tubular damage, which is a potential disadvantage over recombinant Angpt-1 and COMP-Angpt1. In future studies we will investigate VT in a less severe, unilateral ischemia model and compare VT and recombinant Angpt-1 in cultured tubular epithelial cells exposed to hypoxia and inflammatory stimuli.

In summary, we provide proof of principle in support of the efficacious use of PEGylated VT, a drug-like Tie2 receptor agonist, to activate Tie2 *in vivo*, counteract microvascular endothelial barrier dysfunction, improve renal recovery, and reduce mortality in ischemic AKI. Further studies are needed to pave the road for clinical application of this therapy concept.

## Methods

### Vasculotide

Tournaire R. *et al.* previously described the discovery of a short synthetic peptide (HHHRHSF) that binds with high affinity to the extracellular portion of the Tie2 receptor but lacks the capacity to displace either Angpt1 or Angpt2^43^. Using this peptide clustered as a tetramer by way of avidin/biotin, Van Slyke *et al.* demonstrated that Tie2 could be activated in a manner analogous to Angpt1^24^. Subsequently, this proof of principle compound, termed Vasculotide, was reengineered into a more pharmaceutically amenable preparation which excludes the avidin/biotin complex in favor of a 4-armed, polyethylene glycol (average molecular weight 10 kDa) scaffold (Supplementary Fig. S1). A control version of VT was similarly prepared by conjugating 10 kDa PEG (VT backbone) quenched with cysteine on each arm. Synthesis, purification and validation of VT are described in detail elsewhere^23^,^24^.

### *In vivo* animal studies

All procedures were approved by the local committee for care and use of laboratory animals (Landesamt für Natur, Umwelt- und Verbraucherschutz Nordrhein-Westfalen – LANUV) and were performed according to international guidelines on animal experimentation. Eight- to 10-wk-old male C57BL6/J mice (20–25 g) were obtained from Charles River (The Charles River Laboratories; Sulzfeld, Germany). Mice were maintained on mouse chow and tap water ad libitum in a temperature-controlled chamber at 24 °C with a 12:12-h light-dark cycle. Renal IRI in mice was induced by bilateral clamping of the renal pedicles. In brief, mice were anesthetized using isoflurane (induction 3%, maintenance 1.5%, oxygen flow 3 L/minute) and a ventral midline abdominal incision was made. Both renal pedicles were clamped with microvascular clips for 35 min, after that reperfusion was induced by removing the clips. The incision was then closed in layers using 4–0 surgical sutures. Sham animals underwent the same procedure except for clamping of the renal pedicles. Urine and tissues were harvested 24 hours after renal IRI.

### Evans Blue Permeability Assay and wet/dry ratio

24 h after surgery, mice were anesthetized and 1% w/v Evans blue (100 μl) was injected intravenously. Evans blue dye avidly binds to serum albumin and therefore can be used as a tracer for macromolecule flux across the microvasculature. 30 min after application of Evans blue, a blood sample was collected and kidneys were perfused with 10 ml PBS for 2 min via cardiac puncture. Then the kidneys were removed and homogenized in 1.5 ml formamide, followed by incubation at 70 °C for 24 h. The concentration of Evans Blue dye in appropriate dilutions of serum and cleared kidney homogenates was measured spectrophotometrically at 620 nm. The following formula was used to correct the optical densities for contamination with heme pigments: E_620 (corrected)_ = E_620 (raw)_ – (E_405 (raw)_ x 0.014).

For wet/dry ratio, 24 h after IRI or sham operation, kidneys were harvested and weighed (wet weight). After incubation at 70 °C for 24 h, the organs were weighed again (dry weight). Wet/dry ratio is defined as wet weight/dry weight.

### Examination of renal perfusion by CEUS

CEUS examination was performed immediately before and 1 h after surgery, thus every animal served as its own healthy baseline control. In brief, mice were fixed in supine position on a heat plate to prevent hypothermia. Heart rate, body temperature and respiratory rate were monitored throughout the whole examination. Both kidneys were visualized and the most accessible position was chosen to perform the study. A tripod was used to fix the ultrasound transducer (MS 250 Nonlinear Contrast Imaging transducer, VisualSonics Inc, Toronto, Canada) in position and to minimize intra-examination variability. Image depth, focus, gain and frame rate were optimized at the beginning of each study and held constant during the examinations. The contrast agent used in this study was MicroMarker™ (VisualSonics Inc, Toronto, Canada), a non-targeted contrast agent, which contains phospholipid-shelled microbubbles 2–3 μm in size, encapsulating a C_4_F_10_/N_2_ gas mixture. A bolus of 2 μl was injected via the tail vein using a syringe pump to ensure a constant infusion rate and pressure. Starting the contrast agent bolus, an image sequence of the bolus perfusion was acquired (18 MHz, 32 dB). After that a destruction-replenishment sequence was performed. Destruction of the microbubbles was performed by applying five ultrasound pulses (flash) of high acoustic power and then the refilling sequence was acquired. To avoid breathing and other movement artefacts, all sequences were acquired using motion correction (sequences during breathing were skipped).

### Data analysis of CEUS measurements

The Vevo 2100 ultrasound imaging system (VisualSonics Inc, Toronto, Canada) was used to acquire all images. All sequences were exported and analysed with the VevoCQ Contrast Quantification Software (VisualSonics Inc, Toronto, Canada),an application designed for quantifying perfusion in small animal models by CEUS imaging. It provides quantitative measurements by computing different perfusion parameters by means of a dedicated curve-fitting algorithm applied on contrast-uptake kinetics (time intensity curves).

After loading the sequences, 4 microvascular regions of interest (ROI) - 2 in the cortex and 2 in the medulla - were captured. Absence of large vessels in ROIs was verified by lack of bubbles after microbubble destruction (flash). The ROIs of the pre-surgery (baseline) examination were copied and pasted in the corresponding post-surgery sequences to ensure that exactly the same regions were examined in one animal pre- and post-surgery. The software then determined for each ROI the peak enhancement (PE), WiR and TTP from the bolus perfusion sequences and the relative blood volume (rBV) and mean transit time (mTT) from the destruction-replenishment sequences (Supplementary Fig. S2). PE is a measure of relative blood volume, while TTP is an absolute time measurement from the beginning of the bolus to the peak of contrast enhancement. WiR is defined as the maximum slope of the curve fit function which comprises both amplitude and time and is another measure of relative blood flow. The rBV is a measure of the blood volume from destruction to whole refilling and mTT is the time which is needed until half of rBV is reached (Table 1). The rBF was calculated as the rBV/mTT ratio. TTP and mTT are measured in seconds while all other parameters are given in arbitrary units (a.u.). In order to minimize inter-animal variability the mean value of the 2 ROIs from each cortex and medulla was calculated and expressed as percentage change of the post-surgery examination compared to the individual baseline study. Relative renal vascular resistance (rRVR) was calculated as follows: rRVR = MAP/rRBF (given in a.u.).

### Measurement of blood pressure and heartrate

Immediately before the first ultrasound examination, average blood pressure and heart rate were measured via tail cuff in each animal (5 measurements) using the mouse blood pressure system MRBP-M01 (IITC Life science, Woodland Hills, CA, USA). The procedure was repeated after surgery immediately before the second ultrasound examination.

### Clinical Chemistry

Serum creatinine and BUN were measured on an Advia 1800 automated analyzer system (Siemens Healthcare Diagnostics, Eschborn). Creatinine was measured based on the enzymatic reaction of Tanganelli *et al.* (1982). The method was validated by regular analyses of reference sera supplied by the national German INSTAND proficiency testing program and the international quality assurance program of the US Centers for Disease Control and Prevention. The enzymatic creatinine method is traceable to an HPLC candidate reference method, which uses reference materials from the National Institute of Standards and Technology. Urinary levels of NGAL were detected using a specific ELISA kit (BioPorto Diagnostics, Hellrup, Denmark) according to the manufacturer’s instructions.

### Immunofluorescence

Immunofluorescence (IF) was performed on ice cold acetone-fixed cryosections (6 μm) using the following primary antibodies: polyclonal rabbit anti-mouse VE-cadherin Ab (Santa Cruz, Biotechnology, Dallas, Texas, USA), monoclonal rat anti-mouse PECAM-1 (CD31) Ab (BD Biosciences, Franklin Lakes, New Jersey, USA). Nonspecific binding sites were blocked with 10% normal donkey serum (Jackson ImmunoResearch Laboratories, PA, USA) for 30 minutes. Thereafter sections were incubated with the primary antibody (1:200) for 1 h. Both incubations were performed in a humid chamber at room temperature. Slides were washed and the secondary antibodies (goat anti-rabbit Cy3 (Dianova, Hamburg, Germany) and donkey anti-rat alexa fluor 488 (Invitrogen, Carlsbad, CA, USA)) were applied overnight at 4 C (1:1000). For negative controls the staining procedure was performed as described without the primary antibodies. Data were acquired on a Zeiss AxioImager (20x/0.45 objective) equipped with a Hamamatsu ORCA–ER camera. Images were analyzed using Volocity software (ImproVision, Forchheim, Germany).

### Western Blot analysis

For the Western Blot, kidneys of the mice were stored at −80 °C and were directly collected in 1 x Laemmli (4% SDS, 5% 2-mercaptoethanol, 10% glycerol, 0.002% bromophenol blue, 0.0625 M Tris-HCl (pH 6.8) in a tissue grinder. After homogenization of the tissue the samples were heated for 5 min at 95 °C. The samples were then pushed through a 20-gauge-neddle and equal quantities of cell lysates were separated on SDS-PAGE gels. Proteins were transferred to a polyvinylidenfluoride (PVDF) membrane (Merck Millipore, Darmstadt, Germany) and incubated for 30 min at 37 °C in blocking buffer (5% bovine serum albumin (BSA) dissolved in tris-buffered saline (TBS) containing 0.05% Tween-20 (TBS-T). ß-Tubulin served as loading control. Primary antibodies (anti-Tie2 (C-20), Santa Cruz Biotechnology, Dallas, Texas, USA; anti-phospho-Tie2 (Y992), R&D Systems, Minneapolis, MN, USA) were incubated (1:1000 in 5% BSA dissolved in TBS-T) for 1 h at room temperature. Binding of primary antibodies was detected using horseradish peroxidase conjugated secondary antibodies (Dianova, Hamburg, Germany) with a chemiluminescence detection reagent (Roche, Rotkreuz, Switzerland) exposed to a medical X-ray film (Fujifilm, Tokio, Japan).

### Histology

Paraffin-embedded kidney sections were stained with hematoxylin-eosin. Tubular injury was scored by estimating the percentage of tubules in the cortex or the outer medulla that showed epithelial necrosis or had luminal necrotic debris and tubular dilatation as follows: 0 = none; 1 = < 5%; 2 = 5–25%; 3 = 25–75%; and 4 = > 75%.

### Transmission Electron Microscopy

Kidney tissue samples were fixed for 12 h at 4 °C in 3% cacodylate-buffered glutaraldehyde (pH 7.35) and then transferred into 5% sucrose for electron microscopy. Post fixation was performed with 1% osmium tetroxide and 50 mm potassium ferricyanide. Specimens were then washed with distilled water, dehydrated in graded alcohols, and embedded in araldite resin. The ultrathin sections (50 nm) were cut and placed on copper grids. The sections were stained with 5% uranyl acetate and 0.2% lead citrate. Transmission electron microscopic examination was performed using a Philips CMlO electron microscope (Philips, Eindhoven, The Netherlands) operating at 80 kV.17.

### Statistical analysis

Data are presented as means ± standard error of mean (SEM). Differences between groups were compared by one-way analysis of variance (ANOVA) with Tukey as *post hoc* test for multiple comparisons. Kaplan-Meier plots were used to illustrate survival between treatment groups and statistical assessment was performed by the log-rank test. Animals still alive at 14 days after IRI were censored at day 14. All tests were two-sided and significance was accepted at p < 0.05. GraphPad Prism Version 5.02 (GraphPad Prism Software Inc, San Diego, California, USA) was used for data analysis and figure preparation.

## Additional Information

**How to cite this article**: Rübig, E. *et al.* The Synthetic Tie2 Agonist Peptide Vasculotide Protects Renal Vascular Barrier Function In Experimental Acute Kidney Injury. *Sci. Rep.* **6**, 22111; doi: 10.1038/srep22111 (2016).

## Supplementary Material

Supplementary Information

## Figures and Tables

**Table 1:** Hemodynamic variables derived by contrast-enhanced ultrasound (CEUS).

Abbreviation	Parameter	Unit
PE	Peak Enhancement – relative blood volume (plateau value of a curve fit algorithm)	[a.u.]
TTP	Time to peak	[s]
WiR	Wash-in rate (maximum slope) – relative blood flow	[a.u.]
rBV	Relative blood volume	[a.u.]
mTT	Mean transit time	[s]
rBF	Relative blood flow (rBV/mTT)	[a.u.]
rRVR	Relative renal vascular resistance (MAP/rBF)	[a.u.]

PE, TTP and WiR are calculated by the bolus perfusion model, rBV and mTT are calculated using the destruction-replenishment model. rBF is calculated from the fitted curve using extrapolated data. rRVR is calculated as MAP/rBF. PE, WiR, rBV rBF and RVR are given in arbitrary units (a.u.), TTP and mTT in seconds (s).

**Table 2:** Systemic hemodynamics before and 1 h after renal IRI or sham operation.

	sham	PEG IRI	VT IRI
MAP pre surgery [mmHg]	106 ± 11	107 ± 8	104 ± 8
MAP post surgery [mmHg]	111 ± 6	107 ± 8	104 ± 7
HR pre surgery [bpm]	383 ± 14	390 ± 20	392 ± 16
HR post surgery [bpm]	384 ± 17	394 ± 15	399 ± 12

bpm: beats per minute; MAP: mean arterial pressure. Data are presented as means ± SD.
