# Supplementary material for: The Synthetic Tie2 Agonist Peptide Vasculotide Protects Renal Vascular Barrier Function In Experimental Acute Kidney Injury
Source: Sci Rep. 2016 Feb 25;6:22111. doi: 10.1038/srep22111 (PMC4766468; doi:10.1038/srep22111)
Supplement: Supplementary Information [file srep22111-s1.pdf]

Supplementary Information

The Synthetic Tie2 Agonist Peptide Vasculotide Protects Renal Vascular Barrier Function In Experimental Acute Kidney Injury.

Eva Rübig, Jörg Stypmann, Paul Van Slyke, Daniel J Dumont, Tilmann Spieker, Konrad Buscher, Stefan Reuter, Tobias Goerge, Hermann Pavenstädt, Philipp Kümpers

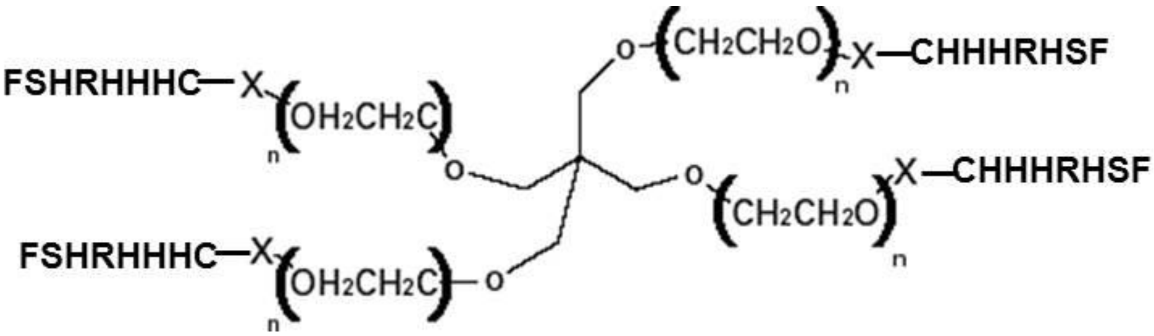

Supplementary Figure S1: Schematic structure of Vasculotide. Four, eight amino acid peptides (NH2-CHHHRHSF-COOH) are covalently attached via cysteine and maleimide (denoted by X) to a 10kDa, tetrameric polyethylene glycol.

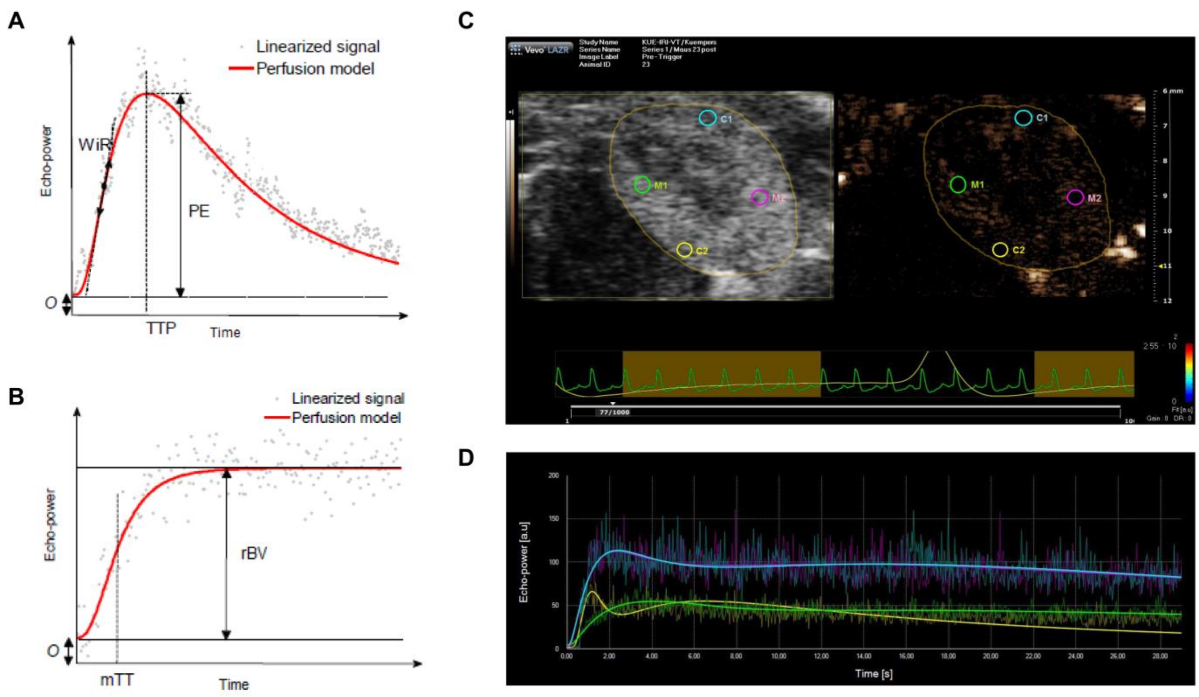

Supplementary Figure S2: Quantification of renal perfusion. **(A)** Bolus perfusion model and **(B)** Destruction**-**Replenishment model curve fit algorithms showing the calculated parameters: Peak enhancement (PE), wash-in rate (WiR), time to peak (TTP), relative blood volume (rBV), mean transit time (mTT). **(C)** Screen shot of the bolus injection showing the whole kidney and the user-defined regions of interest (ROIs) in the cortex (C1+2) and medulla (M1+2) and **(D)** corresponding curves showing the contrast enhancement in the different ROIs. To avoid artefacts, the breathing movement sequences are skipped (the sequences which are used are shown by the light brown bar).

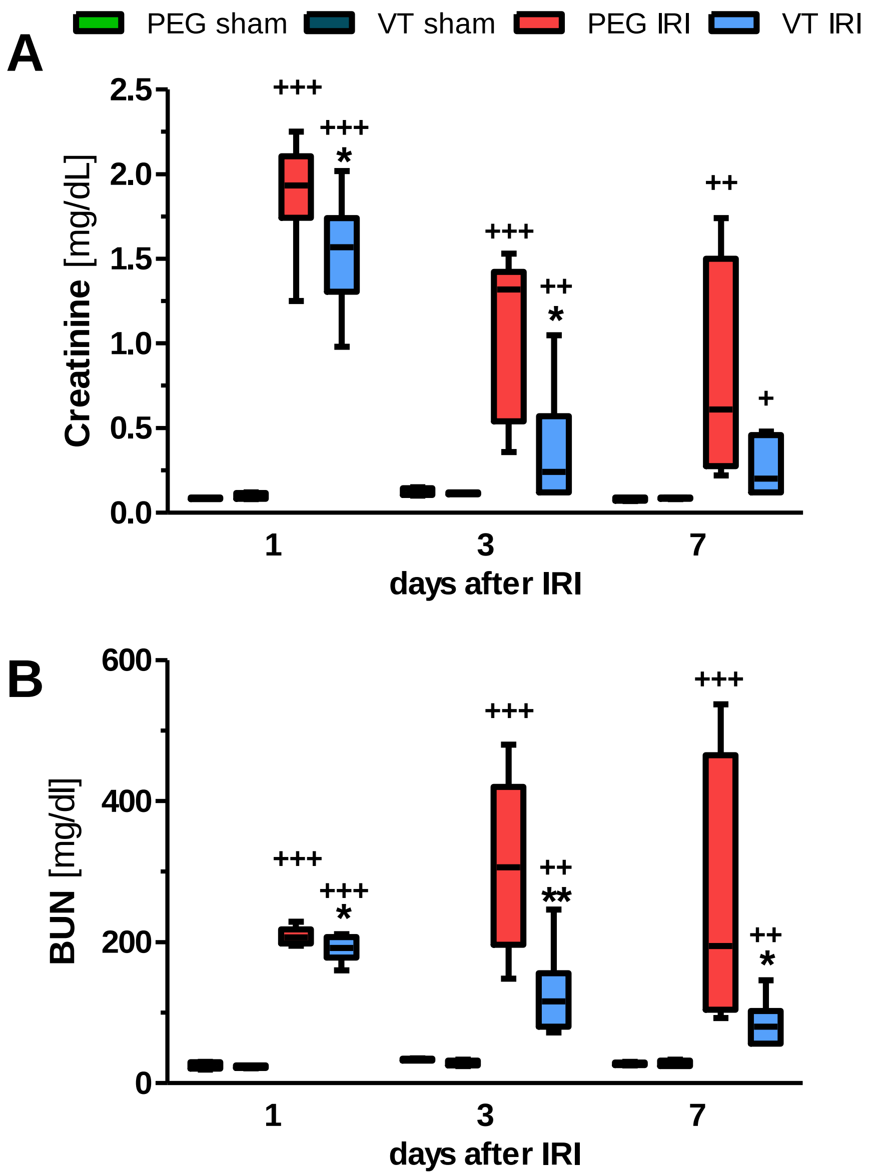

Supplementary Figure S3: Vasculotide improves kidney function and reduces mortality after renal ischemia reperfusion injury. Serum levels of **(A)** creatinine and **(B)** blood urea nitrogen (BUN) at day 1, 3 and 7 after renal ischemia reperfusion injury (IRI – n = 8 per group) or sham surgery (n = 4 per group). Data are expressed as box-and-whisker plot. $*P < 0.05$; $**P < 0.01$ vs. IRI + PEG-Cys. $+P < 0.05$; $++P < 0.01$; $+++P < 0.001$ vs. sham.

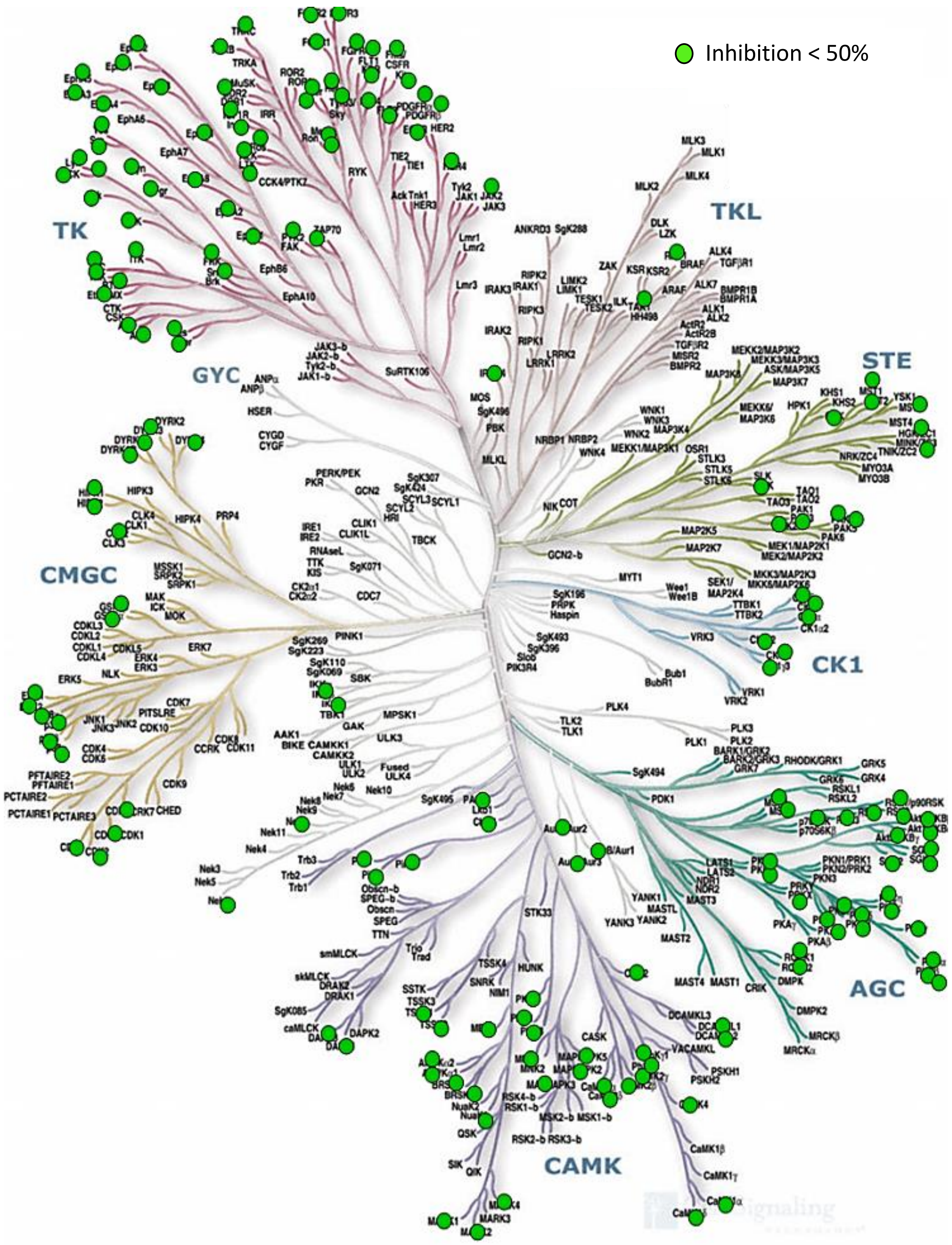

Supplementary Figure S4: Kinome selectivity map. VT does not block the activity of any of the tested 192 human kinases at 10μM concentration. Inhibition < 50% is not considered to represent significant effects of the test compound.

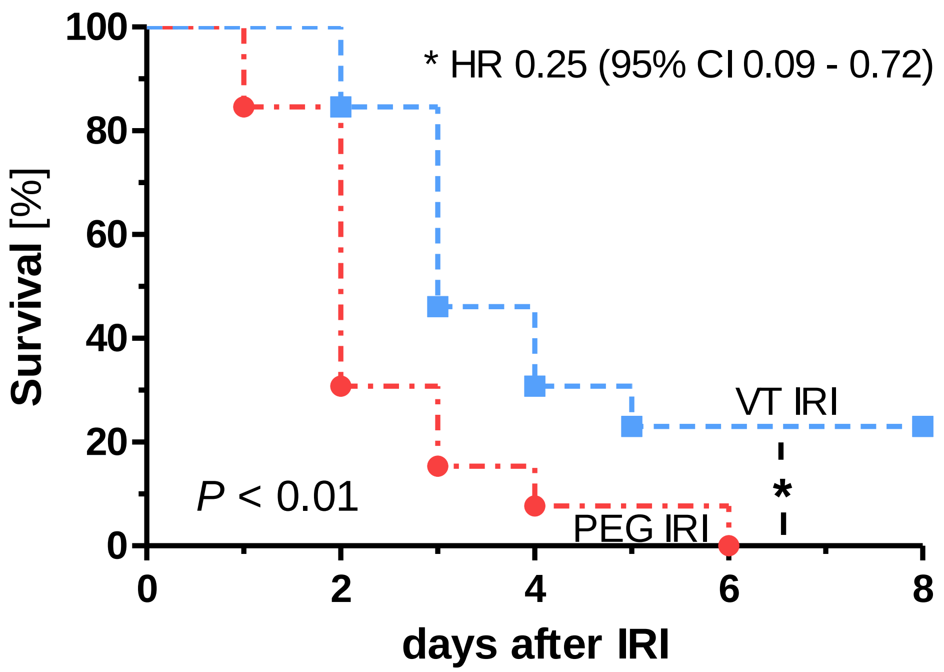

Supplementary Figure S5: Administration of Vasculotide after renal ischemia reperfusion injury reduces mortality. Survival after treatment with 200 ng Vasculotide (VT) or PEG-Cys (PEG) at +2h and +24h *after* renal reperfusion injury (IRI - n = 13 per group). Creatinine and BUN were not measured. Although we did observe a comparable protective effect in the rescue setting too [HR 0.25 (95% CI 0.09 – 0.72)], overall mortality was higher than in the pre-treated groups. We assume that main procedural confounder accounting for this is a different hydration status at the time of the operation, because each injection of VT was solved in 200µl saline. When extrapolated to a human standard body weight of 75 kg, pre- but not rescue treatment therefore included hydration with approximately 1-1.5 liters. HR: Hazard Ratio, CI: Confidence Interval.

Supplementary Table 1: Effects of VT (here EX00120961) and PEG-Cys (EX00120964) in various *in vitro* receptor binding assays.

Assay<br>Cerep Compound I.D.	Client Compound I.D.	Test Concentration (M)	% Inhibition of Control Specific Binding
A_1 *(h)* (antagonist radioligand)			
9770174-1	EX00120964	1.0E-05	-2
9770174-2	EX00120961	1.0E-05	5
A_{2A} *(h)* (agonist radioligand)			
9770174-1	EX00120964	1.0E-05	6
9770174-2	EX00120961	1.0E-05	-7
A_3 *(h)* (agonist radioligand)			
9770174-1	EX00120964	1.0E-05	5
9770174-2	EX00120961	1.0E-05	-11
α_{1A} (antagonist radioligand)			
9770174-1	EX00120964	1.0E-05	-2
9770174-2	EX00120961	1.0E-05	-6
α_{2A} *(h)* (antagonist radioligand)			
9770174-1	EX00120964	1.0E-05	8
9770174-2	EX00120961	1.0E-05	4
β_1 *(h)* (agonist radioligand)			
9770174-1	EX00120964	1.0E-05	-4
9770174-2	EX00120961	1.0E-05	-25
β_2 *(h)* (agonist radioligand)			
9770174-1	EX00120964	1.0E-05	-13
9770174-2	EX00120961	1.0E-05	-9
AT_1 *(h)* (antagonist radioligand)			
9770174-1	EX00120964	1.0E-05	-2
9770174-2	EX00120961	1.0E-05	4
AT_2 *(h)* (agonist radioligand)			
9770174-1	EX00120964	1.0E-05	13
9770174-2	EX00120961	1.0E-05	14
BZD (central) (agonist radioligand)			
9770174-1	EX00120964	1.0E-05	15
9770174-2	EX00120961	1.0E-05	19
BZD (peripheral) (antagonist radioligand)			
9770174-1	EX00120964	1.0E-05	-3
9770174-2	EX00120961	1.0E-05	5
BB (non-selective) (agonist radioligand)			
9770174-1	EX00120964	1.0E-05	-30
9770174-2	EX00120961	1.0E-05	-50
B_1 *(h)* (agonist radioligand)			
9770174-1	EX00120964	1.0E-05	2
9770174-2	EX00120961	1.0E-05	1
B_2 *(h)* (agonist radioligand)			
9770174-1	EX00120964	1.0E-05	-5
9770174-2	EX00120961	1.0E-05	1
CB_1 *(h)* (agonist radioligand)			
9770174-1	EX00120964	1.0E-05	-1
9770174-2	EX00120961	1.0E-05	21
CB_2 *(h)* (agonist radioligand)			
9770174-1	EX00120964	1.0E-05	3
9770174-2	EX00120961	1.0E-05	9
CCK_1 (CCK_A) *(h)* (agonist radioligand)			
9770174-1	EX00120964	1.0E-05	40
9770174-2	EX00120961	1.0E-05	16
CCK_2 (CCK_B) *(h)* (agonist radioligand)			
9770174-1	EX00120964	1.0E-05	-1
9770174-2	EX00120961	1.0E-05	0

Assay<br>Cerep Compound I.D.	Client Compound I.D.	Test Concentration (M)	% Inhibition of Control Specific Binding
**D_1 *(h)* (antagonist radioligand)**			
9770174-1	EX00120964	1.0E-05	0
9770174-2	EX00120961	1.0E-05	4
**D_{2S} *(h)* (antagonist radioligand)**			
9770174-1	EX00120964	1.0E-05	-3
9770174-2	EX00120961	1.0E-05	3
**ET_A *(h)* (agonist radioligand)**			
9770174-1	EX00120964	1.0E-05	1
9770174-2	EX00120961	1.0E-05	9
**$GABA_{A1}$ *(h)* ($\alpha1,\beta2,\gamma2$) (agonist radioligand)**			
9770174-1	EX00120964	1.0E-05	11
9770174-2	EX00120961	1.0E-05	7
**$GABA_{B(1b)}$ *(h)* (antagonist radioligand)**			
9770174-1	EX00120964	1.0E-05	7
9770174-2	EX00120961	1.0E-05	-1
**GAL_1 *(h)* (agonist radioligand)**			
9770174-1	EX00120964	1.0E-05	-4
9770174-2	EX00120961	1.0E-05	-18
**GAL_2 *(h)* (agonist radioligand)**			
9770174-1	EX00120964	1.0E-05	4
9770174-2	EX00120961	1.0E-05	-3
**kainate (agonist radioligand)**			
9770174-1	EX00120964	1.0E-05	1
9770174-2	EX00120961	1.0E-05	45
**glycine (strychnine-sensitive) (antagonist radioligand)**			
9770174-1	EX00120964	1.0E-05	2
9770174-2	EX00120961	1.0E-05	-8
**CXCR2 (IL-8B) *(h)* (agonist radioligand)**			
9770174-1	EX00120964	1.0E-05	17
9770174-2	EX00120961	1.0E-05	12
**CCR1 *(h)* (agonist radioligand)**			
9770174-1	EX00120964	1.0E-05	1
9770174-2	EX00120961	1.0E-05	2
**TNF-α *(h)* (agonist radioligand)**			
9770174-1	EX00120964	1.0E-05	28
9770174-2	EX00120961	1.0E-05	-11
**CCR2 *(h)* (agonist radioligand)**			
9770174-1	EX00120964	1.0E-05	-8
9770174-2	EX00120961	1.0E-05	-1
**H_1 *(h)* (antagonist radioligand)**			
9770174-1	EX00120964	1.0E-05	-10
9770174-2	EX00120961	1.0E-05	-4
**H_2 *(h)* (antagonist radioligand)**			
9770174-1	EX00120964	1.0E-05	-6
9770174-2	EX00120961	1.0E-05	3
**H_3 *(h)* (agonist radioligand)**			
9770174-1	EX00120964	1.0E-05	7
9770174-2	EX00120961	1.0E-05	-7
**H_4 *(h)* (agonist radioligand)**			
9770174-1	EX00120964	1.0E-05	-2
9770174-2	EX00120961	1.0E-05	-1
**MCH_1 *(h)* (agonist radioligand)**			
9770174-1	EX00120964	1.0E-05	-35
9770174-2	EX00120961	1.0E-05	**51**
**MC_3 *(h)* (agonist radioligand)**			
9770174-1	EX00120964	1.0E-05	8
9770174-2	EX00120961	1.0E-05	-8
**MC_4 *(h)* (agonist radioligand)**			
9770174-1	EX00120964	1.0E-05	14
9770174-2	EX00120961	1.0E-05	15

Assay<br>Cerep Compound I.D.	Client Compound I.D.	Test Concentration (M)	% Inhibition of Control Specific Binding
**M_1 *(h)* (antagonist radioligand)**			
9770174-1	EX00120964	1.0E-05	-7
9770174-2	EX00120961	1.0E-05	-6
**M_2 *(h)* (antagonist radioligand)**			
9770174-1	EX00120964	1.0E-05	-12
9770174-2	EX00120961	1.0E-05	-10
**M_3 *(h)* (antagonist radioligand)**			
9770174-1	EX00120964	1.0E-05	-13
9770174-2	EX00120961	1.0E-05	-1
**NK_1 *(h)* (agonist radioligand)**			
9770174-1	EX00120964	1.0E-05	-7
9770174-2	EX00120961	1.0E-05	12
**NK_2 *(h)* (agonist radioligand)**			
9770174-1	EX00120964	1.0E-05	12
9770174-2	EX00120961	1.0E-05	4
**NK_3 *(h)* (antagonist radioligand)**			
9770174-1	EX00120964	1.0E-05	-9
9770174-2	EX00120961	1.0E-05	-12
**Y_1 *(h)* (agonist radioligand)**			
9770174-1	EX00120964	1.0E-05	18
9770174-2	EX00120961	1.0E-05	-10
**Y_2 *(h)* (agonist radioligand)**			
9770174-1	EX00120964	1.0E-05	-1
9770174-2	EX00120961	1.0E-05	8
**NTS_1 (NT_1) *(h)* (agonist radioligand)**			
9770174-1	EX00120964	1.0E-05	1
9770174-2	EX00120961	1.0E-05	2
**NMU2 *(h)* (agonist radioligand)**			
9770174-1	EX00120964	1.0E-05	-7
9770174-2	EX00120961	1.0E-05	-11
**N neuronal $\alpha 4\beta 2$ *(h)* (agonist radioligand)**			
9770174-1	EX00120964	1.0E-05	6
9770174-2	EX00120961	1.0E-05	2
**N muscle-type *(h)* (antagonist radioligand)**			
9770174-1	EX00120964	1.0E-05	-7
9770174-2	EX00120961	1.0E-05	8
**δ_2 (DOP) *(h)* (agonist radioligand)**			
9770174-1	EX00120964	1.0E-05	2
9770174-2	EX00120961	1.0E-05	25
**κ (KOP) (agonist radioligand)**			
9770174-1	EX00120964	1.0E-05	-3
9770174-2	EX00120961	1.0E-05	-8
**μ (MOP) *(h)* (agonist radioligand)**			
9770174-1	EX00120964	1.0E-05	6
9770174-2	EX00120961	1.0E-05	17
**NOP (ORL1) *(h)* (agonist radioligand)**			
9770174-1	EX00120964	1.0E-05	-2
9770174-2	EX00120961	1.0E-05	9
**PCP (antagonist radioligand)**			
9770174-1	EX00120964	1.0E-05	9
9770174-2	EX00120961	1.0E-05	-17
**P2X (agonist radioligand)**			
9770174-1	EX00120964	1.0E-05	-8
9770174-2	EX00120961	1.0E-05	12
**P2Y (agonist radioligand)**			
9770174-1	EX00120964	1.0E-05	-3
9770174-2	EX00120961	1.0E-05	8
**$5\text{-}HT_{1A}$ *(h)* (agonist radioligand)**			
9770174-1	EX00120964	1.0E-05	12
9770174-2	EX00120961	1.0E-05	-8

Assay<br>Cerep Compound I.D.	Client Compound I.D.	Test Concentration (M)	% Inhibition of Control Specific Binding
**$5\text{-}HT_{1D}$ (agonist radioligand)**			
9770174-1	EX00120964	1.0E-05	-6
9770174-2	EX00120961	1.0E-05	-8
**$5\text{-}HT_{2A}$ *(h)* (antagonist radioligand)**			
9770174-1	EX00120964	1.0E-05	-46
9770174-2	EX00120961	1.0E-05	-22
**$5\text{-}HT_{2B}$ *(h)* (antagonist radioligand)**			
9770174-1	EX00120964	1.0E-05	8
9770174-2	EX00120961	1.0E-05	7
**$5\text{-}HT_{2C}$ *(h)* (antagonist radioligand)**			
9770174-1	EX00120964	1.0E-05	5
9770174-2	EX00120961	1.0E-05	2
**$5\text{-}HT_3$ *(h)* (antagonist radioligand)**			
9770174-1	EX00120964	1.0E-05	-4
9770174-2	EX00120961	1.0E-05	1
**$5\text{-}HT_{4e}$ *(h)* (antagonist radioligand)**			
9770174-1	EX00120964	1.0E-05	-10
9770174-2	EX00120961	1.0E-05	-4
**$5\text{-}HT_6$ *(h)* (agonist radioligand)**			
9770174-1	EX00120964	1.0E-05	0
9770174-2	EX00120961	1.0E-05	4
**$5\text{-}HT_7$ *(h)* (agonist radioligand)**			
9770174-1	EX00120964	1.0E-05	4
9770174-2	EX00120961	1.0E-05	6
**σ (non-selective) (agonist radioligand)**			
9770174-1	EX00120964	1.0E-05	21
9770174-2	EX00120961	1.0E-05	-13
**sst (non-selective) (agonist radioligand)**			
9770174-1	EX00120964	1.0E-05	-4
9770174-2	EX00120961	1.0E-05	-12
**GR *(h)* (agonist radioligand)**			
9770174-1	EX00120964	1.0E-05	0
9770174-2	EX00120961	1.0E-05	3
**ERα *(h)* (agonist fluoligand)**			
9770174-1	EX00120964	1.0E-05	11
9770174-2	EX00120961	1.0E-05	9
**PR *(h)* (agonist radioligand)**			
9770174-1	EX00120964	1.0E-05	-5
9770174-2	EX00120961	1.0E-05	11
**AR *(h)* (agonist radioligand)**			
9770174-1	EX00120964	1.0E-05	-13
9770174-2	EX00120961	1.0E-05	-6
**TR (TH) (agonist radioligand)**			
9770174-1	EX00120964	1.0E-05	-6
9770174-2	EX00120961	1.0E-05	42
**$VPAC_1$ (VIP_1) *(h)* (agonist radioligand)**			
9770174-1	EX00120964	1.0E-05	-5
9770174-2	EX00120961	1.0E-05	-3
**V_{1a} *(h)* (agonist radioligand)**			
9770174-1	EX00120964	1.0E-05	-12
9770174-2	EX00120961	1.0E-05	19
**V_{1b} *(h)* (agonist radioligand)**			
9770174-1	EX00120964	1.0E-05	-16
9770174-2	EX00120961	1.0E-05	-2
**V_2 *(h)* (agonist radioligand)**			
9770174-1	EX00120964	1.0E-05	-1
9770174-2	EX00120961	1.0E-05	3
**Ca^{2+} channel (L, dihydropyridine site) (antagonist radioligand)**			
9770174-1	EX00120964	1.0E-05	-11
9770174-2	EX00120961	1.0E-05	-18

Assay<br>Cerep Compound I.D.	Client Compound I.D.	Test Concentration (M)	% Inhibition of Control Specific Binding
**Ca^{2+} channel (L, verapamil site) (phenylalkylamine) (antagonist radioligand)**			
9770174-1	EX00120964	1.0E-05	-1
9770174-2	EX00120961	1.0E-05	5
**Ca^{2+} channel (N) (antagonist radioligand)**			
9770174-1	EX00120964	1.0E-05	-35
9770174-2	EX00120961	1.0E-05	17
**K_{ATP} channel (antagonist radioligand)**			
9770174-1	EX00120964	1.0E-05	8
9770174-2	EX00120961	1.0E-05	24
**K_V channel (antagonist radioligand)**			
9770174-1	EX00120964	1.0E-05	-8
9770174-2	EX00120961	1.0E-05	-4
**SK_{Ca} channel (antagonist radioligand)**			
9770174-1	EX00120964	1.0E-05	-17
9770174-2	EX00120961	1.0E-05	-15
**Cl^- channel (GABA-gated) (antagonist radioligand)**			
9770174-1	EX00120964	1.0E-05	-3
9770174-2	EX00120961	1.0E-05	5
**norepinephrine transporter *(h)* (antagonist radioligand)**			
9770174-1	EX00120964	1.0E-05	8
9770174-2	EX00120961	1.0E-05	13
**dopamine transporter *(h)* (antagonist radioligand)**			
9770174-1	EX00120964	1.0E-05	-11
9770174-2	EX00120961	1.0E-05	-7
**GABA transporter (antagonist radioligand)**			
9770174-1	EX00120964	1.0E-05	-13
9770174-2	EX00120961	1.0E-05	3
**5-HT transporter *(h)* (antagonist radioligand)**			
9770174-1	EX00120964	1.0E-05	-8
9770174-2	EX00120961	1.0E-05	10

Inhibition (or stimulation) >50% is considered to represent a significant effect of the test compound. Results showing an inhibition (or stimulation) <25% are not considered significant and mostly attributable to variability of the signal around the control level. Low to moderate negative values are negligible.

Supplementary Table 2: Effects of VT (here EX00120961) and PEG-Cys (EX00120964) in various *in vitro* enzyme assays.

Assay<br>Cerep Compound I.D.	Client Compound I.D.	Test Concentration (M)	% Inhibition of Control Values
**COX_1 *(h)***			
9770174-1	EX00120964	1.0E-05	-1
9770174-2	EX00120961	1.0E-05	-27
**COX_2 *(h)***			
9770174-1	EX00120964	1.0E-05	19
9770174-2	EX00120961	1.0E-05	8
**12-lipoxygenase *(h)***			
9770174-1	EX00120964	1.0E-05	1
9770174-2	EX00120961	1.0E-05	3
**constitutive NOS *(h)* (endothelial)**			
9770174-1	EX00120964	1.0E-05	-18
9770174-2	EX00120961	1.0E-05	-11
**PDE3A *(h)***			
9770174-1	EX00120964	1.0E-05	0
9770174-2	EX00120961	1.0E-05	0
**$PDE4D_2$ *(h)***			
9770174-1	EX00120964	1.0E-05	-9
9770174-2	EX00120961	1.0E-05	1
**ACE *(h)***			
9770174-1	EX00120964	1.0E-05	-15
9770174-2	EX00120961	1.0E-05	9
**cathepsin D *(h)***			
9770174-1	EX00120964	1.0E-05	-12
9770174-2	EX00120961	1.0E-05	-8
**cathepsin L *(h)***			
9770174-1	EX00120964	1.0E-05	-52
9770174-2	EX00120961	1.0E-05	-66
**MMP-1 *(h)***			
9770174-1	EX00120964	1.0E-05	7
9770174-2	EX00120961	1.0E-05	10
**tryptase *(h)***			
9770174-1	EX00120964	1.0E-05	-13
9770174-2	EX00120961	1.0E-05	2
**phosphatase 1B *(h)* (PTP1B)**			
9770174-1	EX00120964	1.0E-05	11
9770174-2	EX00120961	1.0E-05	-4
**CaMK2α *(h)***			
9770174-1	EX00120964	1.0E-05	-5
9770174-2	EX00120961	1.0E-05	-9
**IRK *(h)* (InsR)**			
9770174-1	EX00120964	1.0E-05	1
9770174-2	EX00120961	1.0E-05	8
**PLC**			
9770174-1	EX00120964	1.0E-05	**72**
9770174-2	EX00120961	1.0E-05	41
**acetylcholinesterase *(h)***			
9770174-1	EX00120964	1.0E-05	34
9770174-2	EX00120961	1.0E-05	-1
**MAO-A *(h)***			
9770174-1	EX00120964	1.0E-05	-8
9770174-2	EX00120961	1.0E-05	1
**MAO-B *(h)***			
9770174-1	EX00120964	1.0E-05	-11
9770174-2	EX00120961	1.0E-05	-6

Inhibition (or stimulation) >50% is considered to represent a significant effect of the test compound. Results showing an inhibition (or stimulation) <25% are not considered significant and mostly attributable to variability of the signal around the control level. Low to moderate negative values are negligible.
